# Cancer survivorship in hematologic malignancies: Lifestyle changes after diagnosis

**DOI:** 10.1002/cam4.3698

**Published:** 2021-02-02

**Authors:** Pannaga Malalur, Manas Agastya, Sandhya Wahi‐Gururaj, Chad L. Cross, Delva Deauna‐Limayo, Edwin C. Kingsley

**Affiliations:** ^1^ Department of Internal Medicine University of Nevada Las Vegas School of Medicine Las Vegas NV USA; ^2^ Division of Medical Oncology The Ohio State University/Wexner Medical Center Columbus OH USA; ^3^ Division of Medical Oncology University of Arizona College of Medicine Tucson AZ USA; ^4^ School of Public Health University of Nevada Las Vegas NV USA; ^5^ VA Southern Nevada Healthcare System Las Vegas NV USA; ^6^ Division of Hematology MountainView Hospital Las Vegas NV USA; ^7^ Comprehensive Cancer Centers of Nevada Las Vegas NV USA

**Keywords:** cancer survivorship, diet, hematologic malignancies, Lifestyle changes, smoking

## Abstract

**Background:**

Studies show that patients make lifestyle changes soon after certain solid tumor diagnoses, suggesting that this may be a teachable moment to motivate and promote healthy behaviors. There is a paucity of data regarding changes made after a diagnosis of a hematologic malignancy.

**Methods:**

A cross‐sectional study of 116 patients at a community oncology center who completed anonymous questionnaires was performed. Questions addressed lifestyle choices made with respect to smoking, alcohol consumption, recreational drug use, diet, and exercise habits before and after diagnosis of a hematologic malignancy. Support systems utilized, including psychiatry services, were also assessed.

**Results:**

Patients exhibited significant reduction in smoking behavior (*Χ* ^2^ = 31.0, *p* < 0.001). 82.4% (*n* = 14) of one pack per day smokers quit between the time periods, with nearly all smokers showing a reduction after diagnosis. Alcohol use overall did not change significantly, however, 10.3% (*n* = 12) of patients reported quitting drinking completely between time periods. Changes in dietary intake and exercise were not statistically significant overall. Utilization of external support systems correlated with improved diet as well as decrease in total smoking years.

**Conclusions:**

This study demonstrates that patients exhibited significant lifestyle changes after being diagnosed with a hematologic malignancy. Clinicians should take advantage of this ‘teachable moment’ to educate patients about positive health behavior changes. Advances in cancer therapeutics have led to an increase in cancer survivors, this education is crucial in reducing the risk of developing chronic comorbidities as well as secondary malignancies.

## Graphical abstract

Patients exhibit significant lifestyle changes after being diagnosed with a hematologic malignancy. Clinicians should take advantage of this “teachable moment” at the time of diagnosis to educate patients about positive health behavior changes.

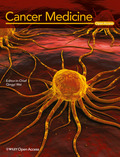

## 1 INTRODUCTION

With the advent of various effective and feasible treatment options for hematologic malignancies, survival rates and quality of life have improved significantly over the last few decades.^1^ Cancer survivors have been shown to be at increased risk of developing chronic comorbidities such as diabetes, obesity, and cardiovascular disease.^2^ Metabolic syndrome associated diseases, particularly obesity, have been associated with increased risk of developing secondary cancers (solid tumors such as breast, colon, renal cell carcinoma, endometrial, adenocarcinoma of the esophagus).^2^ Lifestyle modifications to change detrimental behaviors such as smoking, alcohol consumption, unhealthy diets, and sedentary lifestyle among individuals who have been diagnosed with cancer may help to improve outcomes, or to reduce the incidence of developing negative health outcomes related to unhealthy lifestyle behaviors. For example, healthier choices have the potential to improve prognosis, reduce sequelae of cancer treatments, lower risk of cancer recurrence, decrease incidence of a second cancer, and/or reduce risk for chronic comorbidities such as cardiovascular disease, diabetes, hypertension, and obesity.^2^, ^3^, ^4^

Some studies show that cancer survivors make health behavior changes (e.g., quitting smoking, improving diet and exercise) soon after diagnosis or treatment, suggesting that a cancer diagnosis may motivate and promote healthy behaviors.^3^, ^4^, ^5^, ^6^, ^7^, ^8^, ^9^ The American Cancer Society (ACS) has published guidelines and principles regarding nutrition, physical activity, and alcohol consumption. An ACS study of cancer survivors has demonstrated that a cancer diagnosis could be a “teachable moment” – an opportunity linking a health message to a presenting problem, regardless of the receptivity of the patient.^2^

Strong support systems empower patients to cope with the stress and anxiety of diagnosis and treatment. Patients who demonstrated positive coping behaviors and those who perceived more social support were more likely to exhibit positive health behavior changes.^10^ This is evidenced by studies reporting an association with higher risk health behaviors in cancer survivors who had higher levels of life stressors. Better outcomes were noted when patients took an active approach to coping strategies compared to those who took a passive or indifferent stance.^11^

Much literature on understanding lifestyle changes comes from studying female breast cancer survivors.^7^, ^8^, ^12^, ^13^ Breast cancer survivors have identified family, friends, support groups, and spirituality as sources of social support, enabling them to cope favorably with the negative physical and psychological aspects of breast cancer.^8^ A cross‐sectional study of breast cancer survivors also demonstrated a positive association between active coping strategies and improved diet and exercise patterns.^12^

There is, however, little information available regarding lifestyle changes that patients make after being diagnosed with a hematologic malignancy. The aim of our study was to compare lifestyle modifications (with respect to smoking, alcohol consumption, recreational drug use, diet, and exercise habits) in patients made before and after diagnosis of a hematological malignancy. We also examined support systems utilized and barriers to change identified by our patient population.

## 2 METHODS

The survey instrument was developed based on the literature studying lifestyle changes in female breast cancer survivors and the authors’ clinical experience caring for patients with hematologic malignancies. The questions addressed lifestyle choices made with respect to smoking, alcohol consumption, recreational drug use, diet, and exercise habits before and after diagnosis of a hematologic malignancy. We also inquired about patient demographics and support systems utilized, including psychiatry/mental health services. Behavioral patterns were collected as number of days per week the patient partook in the behavior. We measured fruit days per week as the number of days per week the patient consumed at least 4–5 servings of fruit; cereal days per week as the number of days per week the patient consumed at least six servings of whole grain cereals; low‐fat days as the number of days per week the patient ingested two or fewer servings of fats and oils; and exercise days as the number of days per week the patient participated in at least 30 minutes of exercise/activity. Two open ended questions were also included to understand potential reasons underlying the lifestyle choices that were made.

We surveyed adult patients with a diagnosis of a hematologic malignancy at a community oncology center. Adult patients aged 18 and older with a diagnosis of a hematologic malignancy (i.e., leukemia, lymphoma, or myeloma) either currently undergoing treatment or having completed treatment were included. To assess any differences between patients treated for aggressive vs indolent malignancies, further categorization was made. Patients with CLL, CML, and HCL were grouped as indolent, patients with NHL, HL, and MM as intermediate, and those with AML, ALL, T‐cell leukemia, and DLBCL as aggressive, The survey instrument also required patients to self‐report their current treatment status (ongoing, completed, or never requiring treatment). Patients with multiple malignancies, incomplete surveys, and protected populations (such as pregnant or incarcerated patients) were excluded.

Clinic staff provided survey forms to all patients who were willing to participate in the study. Completed surveys were returned by the patients in sealed envelopes which were immediately placed in a locked container to maintain anonymity. No protected health information was collected on the surveys. Members of the research team reviewed and compiled data from the anonymous surveys.

### 2.1 Statistical methods

Data analyses were conducted for main effects of interest and for subgroup analyses where appropriate. For comparisons of continuous outcomes, data were first examined for normality using both the Shapiro‐Wilk test and by examination of measures of skewness and kurtosis. In this study, the data met appropriate distributional assumptions, and therefore a paired‐samples *t*‐test was used. Changes in categorical responses were analyzed using the McNemar‐Bowker test with exact *p*‐values. All analyses were conducted using SPSS (v. 24).

This study was approved by the Institutional Review Board of the University of Nevada School of Medicine, Las Vegas (UNLV).

## 3 RESULTS

Of the 139 patients who were approached, nine patients declined to participate in the study, 14 patients were excluded due to incomplete surveys (*n* = 6) and a history of multiple malignancies (*n* = 8), resulting in 116 completed surveys included for data analysis. Patient characteristics are described in detail in Table 1.

**TABLE 1:** Demographic and study sample characteristics

Variable	Category	Frequency (*N*)	Percent (%)
Sex	Female	56	48.3
	Male	60	51.5
Race	African American	12	10.3
	Asian/PI	3	2.6
	Caucasian	72	62.1
	Hispanic	22	19.0
	Other	5	4.3
	Unknown	2	1.7
Marital status	Divorced	19	16.4
	Married	66	56.9
	With partner	3	2.6
	Single/Never Married	16	13.8
	Widowed	12	10.3
Employment	Employed	44	37.9
	Not employed	35	30.2
	Retired	36	31.0
	Unknown	1	0.9
Education	Less than HS	13	11.2
	High school	28	24.1
	College	52	44.8
	Vocational	22	19.0
	Unknown	1	0.9
Income	<30 K	29	25
	30–50 K	23	19.8
	50–100 K	25	21.6
	>100 K	14	12.1
	Unknown	25	21.6
Diagnosis	Leukemia	30	25.9
	Lymphoma	61	52.6
	Myeloma	25	21.6
Years	0–1	23	19.8
Since	2–10	68	58.6
Diagnosis	>10	25	21.6
Prognosis	Aggressive	18	15.5
	Indolent	25	21.5
	Intermediate	70	60.3
	Not reported	3	2.6
Treatment status	Complete	51	44.0
	Current	53	45.7
	Never required	12	10.3

Patients ranged in age from 19 to 89 years, with a median age of 62 years (Mean = 60.3, SD = 15.51). Most patients were Caucasian (62.1%, *n* = 72), married or with a partner (59.5%, *n* = 69), and had a college or vocational degree (63.8%, *n* = 74). Patients were relatively uniformly distributed in terms of employment status and income. Most patients had a diagnosis of lymphoma (52.6%, *n* = 61) and were within 2–10 years since diagnosis (58.6%, *n* = 68). Regarding prognosis, most patients were of intermediate cancer severity (60.3%) with either a complete (44.0%) or current (45.7%) treatment status (Table 1).

### 3.1 Smoking, drug, and alcohol use behavior

Smoking behaviors, as measured by packs per day, changed significantly after a cancer diagnosis (*Χ* ^2^ = 31.0, *p* < 0.001). Most notably, zero nonsmokers initiated smoking behavior after diagnosis, a large percentage of smokers quit entirely (82.4%, *n* = 14, of 1–2 pack per day smokers and 50%, *n* − 2, of >2 pack per day smokers), with nearly all participants who smoked at time one (T1, before diagnosis) reducing their packs per day smoked at time two (T2, after diagnosis). This reduction in smoking also resulted in a significant drop in the total number of reported smoking years for patients between T1 (mean = 20.77, SE = 2.494) and T2 (mean = 16.71, SE = 2.652) resulting in a mean reduction of 4.1 years (*t* = 2.436, *p* = 0.023). Alcohol use, measured as drinks per week, did not change significantly (*Χ* ^2^ = 10.6, *p* = 0.102); however, it is notable that 10.3% (*n* = 12) of patients reported quitting drinking completely between time periods (Table 2). Results in drug and alcohol use as reported above were similar between sexes.

**TABLE 2:** Summary table of changes in drug and alcohol use between Time 1 (before diagnosis) and Time 2 (after diagnosis); percentages are shown based on row totals (*N*)

Number of packs a day	Patients (*N*) at Time 1	Change in behavior, cigarette packs per day at Time 2 for the same patients compared to Time 1				*Χ* ^2^ statistic	*p*‐value
		0	<1	1–2	>2		
Smoking (cigarettes)							
0	71	71 (100%)	0 (0%)	0 (0%)	0 (0%)	31.0	<0.001
<1	21	14 (66.7%)	6 (28.6%)	1 (4.8%)	0 (0%)		
1–2	17	14 (82.4%)	1 (5.9%)	2 (11.8%)	0 (0%)		
>2	4	2 (50%)	0 (0%)	1 (25%)	1 (25%)		

Number of hits a day	Patients (*N*) at Time 1	Change in behavior, marijuana hits per day at Time 2 for the same patients compared to Time 1				*Χ* ^2^ statistic	*p*‐value
		0	1–5	5–10	>10		
Marijuana use							
0	109	109 (100%)	0 (0%)	0 (0%)	0 (0%)	103.1	<0.001
1–5	1	0 (0%)	1 (100%)	0 (0%)	0 (0%)		
5–10	2	0 (0%)	0 (0%)	2 (100%)	0 (0%)		
>10	3	1 (33.3%)	0 (0%)	0 (0%)	2 (66.7%)		

Number of drinks per day	Patients (*N*) at Time 1	Change in behavior, alcoholic drinks per day at Time 2 for the same patients compared to Time 1					*Χ* ^2^ statistic	*p*‐value
		0	<1	1–5	5–7	>7		
Alcohol consumption								
0	61	58 (95.1%)	0 (0%)	3 (4.9%)	0 (0%)	0 (0%)	10.6	0.102
<1	1	0 (0%)	1 (100%)	0 (0%)	0 (0%)	0 (0%)		
1–5	35	7 (20%)	0 (0%)	28 (80%)	0 (0%)	0 (0%)		
5–7	10	1 (10%)	0 (0%)	1 (10%)	8 (80%)	0 (0%)		
>7	9	4 (44.4%)	0 (0%)	2 (22.2%)	1 (11.1%)	2 (22.2%)		

Data were too sparse to test significant differences based on cancer severity and treatment status, but some results were interesting. One patient with aggressive cancer reported smoking >2 packs/day and continued to do so after treatment, but nearly all of the other smokers reported decreases in packs per day at T2. Further, four patients went from multiple drinks per week to 0 reported drinks per week between time periods, and this occurred regardless of cancer severity. THC use mirrored that of the entire sample. Patterns were similar across variables for treatment status, with general decreases between time periods. Interestingly, even those who never received treatment reported smoking cessation.

### 3.2 Diet and exercise

Changes in dietary intake and exercise were not consistent between time periods. Mean fruit days per week increased significantly between time periods (*t* = 3.100, *p* = 0.002). This result was most notable for males (*t* = 3.845, *p* < 0.001), those who reported being employed (*t* = 3.947, *p* < 0.001), those 2–10 years post diagnosis (*t* = 3.204, *p* = 0.002), those with curable cancers (*t* = 2.332, *p* = 0.025), and those with lymphoma (*t* = 3.487, *p* = 0.001). This trend was also true for those with intermediate cancers and those who completed treatment, however was not statistically significant.

Mean cereal days increased slightly, but not significantly, from T1 to T2, whereas low fat diet consumption and exercise days slightly decreased from T1 to T2. Fat consumption also decreased significantly for those with indolent cancers (*t* = 2.500, *p* = 0.019), and those under current treatment (*t* = 2.599, *p* = 0.012) (Table 3).

**TABLE 3:** Means and standard errors for Time 1 (before diagnosis) and Time 2 (after diagnosis) measurements for Diet and Exercise variables. Paired *t*‐tests with exact *p*‐values are reported

Measure	Time 1	Time 2	Mean difference	*t*‐statistic	*p*‐value
Total sample					
Fruit days	3.59 (2.090)	4.15 (2.040)	−0.557	−3.100	0.002
Cereal days	3.68 (2.286)	3.80 (2.154)	−0.114	−0.625	0.534
Fat days	3.46 (2.116)	3.25 (2.151)	0.209	1.468	0.145
Exercise days	3.00 (2.485)	2.92 (2.658)	0.079	0.361	0.719
Cumulative	13.43 (6.446)	14.14 (6.682)	−0.704	−1.260	0.210
Aggressive cancer					
Fruit days	3.56 (1.977)	4.44 (2.036)	−0.889	−1.978	0.064
Cereal days	4.22 (1.896)	4.56 (1.977)	−0.333	−0.669	0.513
Fat days	3.00 (1.910)	3.17 (2.229)	−0.167	−0.402	0.692
Exercise days	2.76 (2.658)	2.41 (2.763)	0.353	0.636	0.534
Cumulative	13.39 (7.163)	14.44 (7.602)	−1.056	−0.629	0.538
Indolent cancer					
Fruit days	3.71 (2.258)	3.68 (2.074)	0.036	0.099	0.922
Cereal days	3.59 (2.454)	3.67 (2.386)	−0.074	−0.189	0.852
Fat days	3.56 (2.172)	3.00 (2.166)	0.566	2.500	0.019
Exercise days	3.37 (2.388)	2.74 (2.379)	0.630	1.885	0.071
Cumulative	13.32 (6.549)	12.75 (6.287)	0.571	0.676	0.505
Intermediate cancer					
Fruit days	3.55 (2.076)	4.26 (2.027)	−0.710	−3.088	0.003
Cereal days	3.58 (2.323)	3.65 (2.092)	−0.072	−0.317	0.752
Fat days	3.54 (2.158)	3.37 (2.148)	0.171	0.909	0.367
Exercise days	2.91 (2.501)	3.11 (2.748)	−0.200	−0.663	0.509
Cumulative	13.49 (6.307)	14.62 (6.607)	−1.130	−1.509	0.136
Treatment complete					
Fruit days	3.38 (1.905)	4.10 (2.023)	−0.720	−2.599	0.012
Cereal days	3.50 (2.197)	3.66 (2.182)	−0.160	−0.610	0.545
Fat days	3.57 (2.229)	3.69 (2.214)	−0.118	−0.465	0.644
Exercise days	3.16 (2.502)	3.52 (2.735)	−0.360	−0.994	0.325
Cumulative	13.42 (6.834)	15.00 (7.332)	−1.580	−1.608	0.114
Treatment current					
Fruit days	3.75 (2.227)	4.19 (2.039)	−0.434	−1.512	0.137
Cereal days	3.88 (2.315)	3.85 (2.052)	0.038	0.133	0.895
Fat days	3.33 (2.007)	2.88 (2.006)	0.442	2.599	0.012
Exercise days	2.77 (2.414)	2.23 (2.438)	0.538	1.678	0.099
Cumulative	13.49 (6.197)	13.19 (6.070)	0.302	0.404	0.688
Treatment never received					
Fruit days	3.75 (2.301)	4.17 (2.290)	−0.417	−2.159	0.054
Cereal days	3.58 (2.644)	4.17 (2.588)	−0.583	−1.134	0.281
Fat days	3.58 (2.234)	3.00 (2.335)	0.583	1.735	0.111
Exercise days	3.33 (2.839)	3.42 (2.746)	−0.083	−1.000	0.339
Cumulative	13.25 (6.398)	14.75 (6.398)	−1.500	−1.807	0.098

### 3.3 Psychiatric visits and external support systems

Interestingly, only 19 patients (16.5%) reported seeing a psychiatrist. Two of these patients reported seeking psychiatric support at T2 even though they did not have a history of seeing a psychiatrist at T1. Of the 17 patients who sought psychiatric treatment at T1, eight (47.1%) stopped seeing the psychiatrist at T2, though this was not significant (exact binomial, *p* = 0.109).

Of further interest is that 50% of those with aggressive cancers reported using four or more support systems compared to only 25% for indolent and 16% for intermediate cancers; also a third of those who completed treatment also used four or more support systems, compared to 17% and 8% for those currently in treatment and those never receiving treatment, respectively. The use of psychiatric treatment did not differ as a function of cancer severity or treatment status (all *p* > 0.05).

Two open‐dialog questions were included in our survey addressing issues patients faced that were either beneficial or detrimental to healthy living. These questions were included in order to identify concepts that require further research. These descriptive data were reviewed to identify common themes expressed by patients. Many patients expressed a heightened awareness of health after their cancer diagnosis. Patients frequently identified challenges such as advancing age, comorbidities (most common were diabetes, chronic pain, chronic kidney disease), lack of familial support, fatigue, reduced appetite, anxiety, stress, and financial burden of healthy choices (e.g., “healthy food is expensive, fast‐food is cheaper and more accessible”) as factors that prevented healthy lifestyle changes. Recurring elements supporting positive change included maintaining a positive attitude, access to educational material/physician counseling, personally researching healthful choices, having long‐term goals, mindfulness, and strong support from family, friends, and/or religion.

## 4 DISCUSSION

Our study demonstrates patients with hematologic malignancies make positive lifestyle and behavior changes, most notably by reducing or quitting smoking. Our findings are comparable to other studies that have shown similar behavioral change patterns after receiving major diagnoses such as cardiovascular disease or solid malignancies.^14^, ^15^

There is a critical need to improve prevention and management of chronic comorbidities in the growing population of cancer survivors. At the time of a serious diagnosis, patients experience an increased emotional state, during which they often realize or better understand the impact of their daily choices. It is in this period that patients are often more open to a positive healthcare message and are more likely to implement major lifestyle changes. It is important that providers are aware of this opportunity and utilize it to motivate patients to adopt or improve healthy behavior and lifestyle choices.^2^, ^15^ Understanding the rationale behind positive and negative changes made by these patients is of great importance as this will enable healthcare staff to identify factors that facilitate or impede positive change. A collaborative model integrating technology to deliver behavioral interventions along with routine survivorship services has been suggested to manage healthcare promotion and care coordination between the primary care provider and specialist.^16^

Our patients identified common themes such as current health status, treatment side effects, social support, finances, motivation, and access to education/research materials as among factors influencing health behavior change. Similar attitudes and challenges have been identified in other patient populations including endometrial cancer survivors^17^ and adolescent/young adult cancer survivors.^18^ Breast cancer patients who felt that they had control over their health tended to improve diet and exercise habits.^13^

Diagnostic interviews and self‐report instruments have revealed high prevalence of depression and psychosocial distress in cancer patients.^19^ The SMaRT Oncology‐2 trial showed that a collaborative treatment approach for major depression in cancer patients with the assistance of psychiatrists, primary care physicians, and nurses resulted in reduced depression, anxiety, fatigue, pain, improved quality of life, overall functioning, and health.^20^ We observed that only a small number of patients in our study utilized the assistance of mental health services. Improving screening for depression at clinic visits and counseling patients regarding the availability and benefit of these services may help to alleviate an unmet need. In addition to psychiatric visits, external support systems may be important for lifestyle changes in these patients. Patients who perceived having more social support were better able to cope with the stresses of a cancer diagnosis and treatment, and were more likely to make positive health behavior changes.^10^ Though sample sizes are often small when doing subanalyses, our study suggests that having external support systems is related to decreased total smoking years (1–3 support systems mean difference = 3.816, *t* = 2.199, *p* = 0.041) and increased fruit days per week (1–3 support systems mean difference = −0.537, *t* = −2.507, *p* = 0.014; 4+ support systems mean difference = −0.963, *t* = −2.871, *p* = 0.008).

We recognize there are some limitations to this study. Some degree of recall bias is anticipated; patients may overestimate the extent of lifestyle modifications made, and may not be entirely truthful about the magnitude of changes implemented. Our sample size was not large enough to perform detailed subgroup analyses. We also did not assess the true impact of the teachable moment on our patients as we did not survey how they were counseled on behavioral modifications at the time of diagnosis. It is also possible that some long‐term survivors may have made short‐lived changes initially that were not sustained and therefore not captured.

Future directions: The initial results of this study serve to generate hypotheses for future research and to underscore the importance of assessing lifestyle behaviors as a critical component of holistic cancer treatment. Providing patients with adequate advice and strong support regarding lifestyle modification, taking advantage of the “teachable moment”, along with close follow‐up at regular intervals to ensure that the positive behaviors are sustained, is of utmost importance. This multidimensional support will ultimately improve the well‐being and quality of life of patients with hematologic malignancies and decrease the burden of chronic comorbidities.

## 5 CONCLUSION

This study demonstrates that patients exhibit mostly positive behavioral changes after diagnosis of a hematologic malignancy. A large percentage of these patients quit smoking completely, and nearly all smokers reduced the amount smoked. Overall, our patients displayed improvements in diet, but not exercise. There was also a trend towards positive behavioral changes in patients with more aggressive malignancies. We observed that only a small number of patients in our study utilized the assistance of mental health services, outlining that this is a clear unmet need. Having external support systems appeared to be related to positive behavior changes.

## CONFLICTS OF INTEREST

All authors claim no conflict of interest.

## AUTHOR CONTRIBUTIONS

Conceptualization: Pannaga Malalur, Sandhya Wahi‐Gururaj, Edwin C. Kingsley. Data Curation: Pannaga Malalur, Manas Agastya, Edwin C. Kingsley. Methodology and statistical analysis: Chad L. Cross. Resources: Sandhya Wahi‐Gururaj, Edwin C. Kingsley, Delva Deauna‐Limayo. Writing –original draft: Pannaga Malalur, Manas Agastya, Sandhya Wahi‐Gururaj. Writing‐review and editing: all authors.

## Data Availability

All data generated or analyzed during this study are included in this published article.
